# Partial-Gated Memristor Crossbar for Fast and Power-Efficient Defect-Tolerant Training

**DOI:** 10.3390/mi10040245

**Published:** 2019-04-13

**Authors:** Khoa Van Pham, Tien Van Nguyen, Kyeong-Sik Min

**Affiliations:** School of Electrical Engineering, Kookmin University, Seoul 02707, South Korea; khoapv@kookmin.ac.kr (K.V.P.); tiennv@kookmin.ac.kr (T.V.N.)

**Keywords:** memristor crossbar, partial-gated, fast and power-efficient training, defect-tolerant training

## Abstract

A real memristor crossbar has defects, which should be considered during the retraining time after the pre-training of the crossbar. For retraining the crossbar with defects, memristors should be updated with the weights that are calculated by the back-propagation algorithm. Unfortunately, programming the memristors takes a very long time and consumes a large amount of power, because of the incremental behavior of memristor’s program-verify scheme for the fine-tuning of memristor’s conductance. To reduce the programming time and power, the partial gating scheme is proposed here to realize the partial training, where only some part of neurons are trained, which are more responsible in the recognition error. By retraining the part, rather than the entire crossbar, the programming time and power of memristor crossbar can be significantly reduced. The proposed scheme has been verified by CADENCE circuit simulation with the real memristor’s Verilog-A model. When compared to retraining the entire crossbar, the loss of recognition rate of the partial gating scheme has been estimated only as small as 2.5% and 2.9%, for the MNIST and CIFAR-10 datasets, respectively. However, the programming time and power can be saved by 86% and 89.5% than the 100% retraining, respectively.

## 1. Introduction

Neural networks can be implemented with memristor crossbars, where the memristors can represent adjustable synaptic connections between neurons [1]. Applying an electrical signal to a memristor can gradually change memristor’s conductance, as the synaptic weight is changed according to a sensory stimulus in the biological neuronal system. Actually, the memristors have been experimentally demonstrated in 2008 [2]. Since then, they have been intensively studied as a possible candidate for implementing neural-networks in nanoscale [2]. Memristor crossbars can be built in three-dimensional architecture, which seems to be very similar to the biological neuronal structure that was observed in mammalian brains [3,4,5]. Moreover, memristor crossbars can be fabricated while using the Back-End-Of-Line process on the top of Silicon substrate [3,4]. Additionally, their non-volatile and non-linear behaviors can be useful in performing cognitive computing with memristor crossbars [6,7].

**Figure 1:**
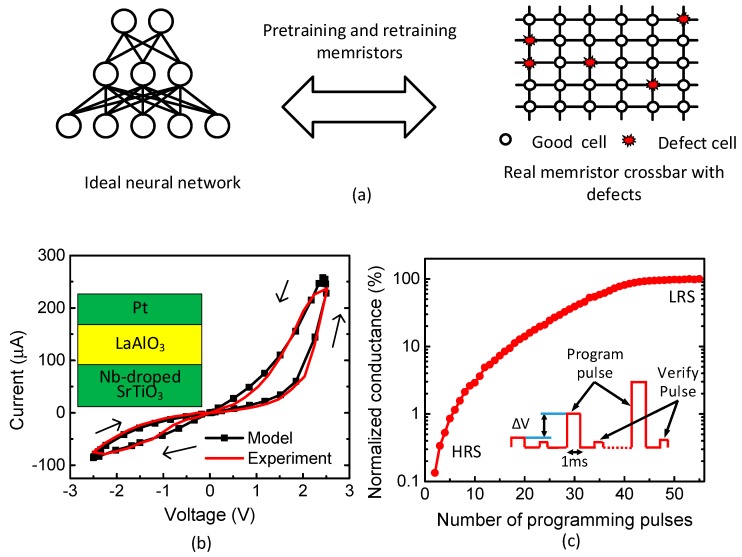
(**a**) The conceptual diagram of the ideal neural network versus the real memristor crossbar with defects; (**b**) the measured memristor’s butterfly curve and the Verilog-A model [11] with the cross-sectional view of the measured memristor [12], adapted from [11,12]; and (**c**) the memrisrtor’s conductance change with increasing the number of programming pulses. The inset figure shows the program-verify scheme of memristor programming based on the ISPP method of Flash memories, adapted from [13]. Vp is the programming voltage increased from 1.1 V to 2.8 V. The programming pulse width is 1 ms.

One thing to consider in implementing the memristor-based neural networks is memristor defects, as shown in Figure 1a. In real crossbars, there are stuck-defects, such as stuck-at-0, stuck-at-1, etc. [8,9]. In addition, we can observe variation-related defects, where each memristor can have different LRS and HRS values [10]. Here, LRS and HRS are Low Resistance State and High Resistance State, respectively. Figure 1a shows a conceptual diagram of the ideal neural network versus a real memristor crossbar with defects. Figure 1b shows a typical butterfly curve of the memristors measured [11,12]. The measured memristor has the film structure of the Pt/LaAlO_3_/Nb-doped SrTiO_3_ stacked layer. The measurement and fabrication of the memristor were explained in the previous publications [11,12]. Here, the LRS and HRS measured are 10 kΩ and 1 MΩ, respectively. The black line in Figure 1b represents the behavioral model of the memristor measured [11,12]. The detailed equations of the memristive behavioral model can be found in the previous publication [11]. The model equations were implemented in the Verilog-A model in CADENCE SPECTRE for the circuit simulation in this paper. 

Figure 1c shows memristor’s conductance change with respect to the number of programming pulses. Here, the inset figure of Figure 1c describes the program-verify scheme, where a verifying pulse to accurately control the memristor’s conductance change follows a programming pulse [11]. Here, the programming voltage amplitude is modulated increasingly to reduce the number of programming pulses, like ISPP (Incremental Step Pulse Programming) used in FLASH memories [13]. The programming and verifying pulses are repeated over until the memristor’s conductance reaches a target value. From Figure 1c, the number of programming pulses is observed as many as 55 for V_P_ = 2.8 V [11]. V_P_ is the amplitude of the programming voltage. In this figure, the memristor’s conductance can reach the target value after 55 programming pulses. The programming pulse width and amplitude can be different, according to memristor materials, etc. [11]. From Figure 1c, the programming time can be calculated with the ‘m’ times of each programming pulse’s width. Here, ‘m’ means the number of pulses needed to reach the target conductance. To reduce the programming time, the number of pulses or the pulse width should be decreased, as indicated in Figure 1c.

As mentioned earlier, the large number ‘m’ of programming pulses results in a long programming time and a large amount of programming power in the memristor crossbar. Thus, to save the memristor programming time and power, in this paper we propose a new partial gating scheme for energy-efficient crossbar training. In the partial gating scheme, only part of neurons that are more responsible for the error are selectively trained, instead of training the entire crossbar. By doing so, we can drastically reduce the programming time and the power of memristors, in this paper.

The energy-efficient and fast training of memristor-based neural networks are very important in edge-computing applications [14]. For edge-computing, the data are processed not by the high-performance cloud servers, but the edge-node devices, where various physical-world data are sensed, collected, and interpreted [15]. For the data type, most of data collected at the edge nodes are unstructured data, like images, voices, anomaly patterns, etc. [14,15]. In order to recognize a vast amount of the unstructured data from the physical world, a cognition algorithm that is inspired by the brain’s architecture and function should be used at the edge devices. If we assume that only the cloud, and not the edge, processes all of these unstructured data, the cloud’s energy should be enormously consumed. Thus, the edge devices become more important, as more data need to be processed at the edge nodes [15,16]. 

Fortunately, the memristor-based neural networks can be easily integrated into the edge-computing devices [15]. To do so, the training power and time of memristor-crossbar networks should be considered as well as the network’s cognitive performance. In this regard, the proposed partial gating scheme will be useful in the future memristor-based neural networks for edge computing.

## 2. Method

### 2.1. The Partial Gating Scheme

**Figure 2:**
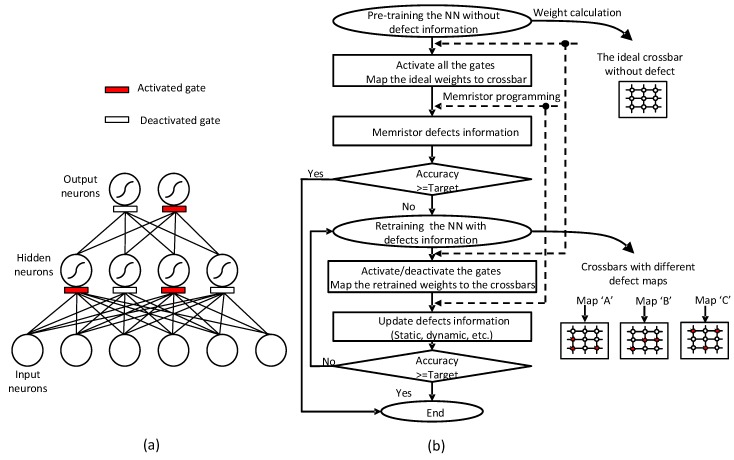
(**a**) The neural network with the partial gating. Here the neurons responsible to the output error are activated and the others related little to the error are deactivated. By doing so, the retraining time can be saved because only the connections belonging to the activated gates are programmed during the retraining. (**b**) The flowchart of training steps of the proposed partial-gated memristor network. During the pre-training, the ideal crossbar is assumed and all of the weights are trained. During the retraining, parts of the gates are activated and only the connections to the gates are retrained. By doing so, we can save a lot of programming time and power during the retraining.

Figure 2a shows a conceptual partial gating scheme that can selectively activate and deactivate the gates of neurons, in order to reduce the programming time and power in training memristor crossbar. In Figure 2a, the network is composed of output, hidden, and input neurons. The activated and deactivated gates during the training are shown in empty and filled patterns, respectively. Instead of activating all the gates, only parts of the gates are activated in Figure 2a, to realize the concept of partial gating.

Figure 2b shows a flowchart of the proposed partial gating scheme for training the memristor crossbar with random-distributed defects. The flowchart is composed of the pre-training and retraining steps. The pre-training is for training the ideal memristor crossbar without defects. During the pre-training, all of the gates are activated and the back-propagation algorithm updates all of the synaptic weights. In the pre-training, all the memristors are programmed according to the calculated weights. After the pre-training, if the recognition rate is still lower than a target performance due to the defects, the retraining should be performed. For doing this, we can use the partial gating scheme. In the partial gating scheme, parts of the gates that are more responsible for the output error are chosen. Subsequently, only the connections belonging to the selected gates are retrained. This retraining step can repeatedly happen, even after the target performance is reached. This is due to the dynamic defects occurring with respect to the operation time. The dynamic defects may be caused by various sources, such as endurance, retention, drift in memristance, migration, etc. [17]. Thus, retraining all of the synaptic weights to recover the loss due to the dynamic defects is very wasteful in terms of programming time and power. By applying the partial gating scheme to the retraining, the programming power and time can be significantly saved, as will be shown in this paper.

To clearly address the difference of the partial gating scheme from the previous techniques, we consider the previous pruning and partial-training techniques here. First, we start from the pruning technique [18,19]. The pruning technique has been used to reduce the size of neural networks by eliminating some insignificant synaptic connections and neurons from the network [18,19]. In the pruning, synaptic weights and neurons in the neural network are ranked based on their impact on recognition performance. Afterwards, the low-ranked nodes and connections are eliminated from the network. By doing so, the network can be compressed to a smaller size. However, in the pruning, the entire network should be trained during the retraining, which is different from the partial gating scheme. The detailed comparison between the pruning and the partial gating schemes will be later explained in Table 2, in terms of recognition rate, training power, and time. The comparison in Table 2 indicates that the pruning technique shows a much worse recognition rate than the partial gating scheme, though the training time and power seem similar, as will be explained later.

The previous partial training was introduced to compensate stuck-defects in the conventional CMOS-based hardware systems [20]. In the previous partial training, if a neuron’s link is defected, such as stuck-at-0 or stuck-at-1, all of the links that belong to the defected neuron should be retrained again [8,9,21]. This kind of previous partial training cannot be used in the memristor crossbar, where many defects are randomly distributed over the entire crossbar [9]. If this previous partial training is used for the memristor crossbar, most of memristors should be retrained every time, because the defects are randomly distributed over the entire network. Thus, we cannot partially train the memristor crossbar by the previous partial training. On the contrary, in this paper, we propose the new mathematical calculation by which the gating scheme can train neurons partially not all, in spite of the randomly-distributed memristor defects, as will be shown in Equations (1)–(3). According to these equations, parts of neurons can be activated during the retraining, if they are more responsible to the output error. The others are deactivated, because they are less responsible. By doing so, the training power and time can be saved in the partial gating scheme.

### 2.2. The Mathematical Equations for the Partial Gating Scheme

Here, we explain the partial training algorithm. The gates can be activated or deactivated in the memristor network according to the output error. If some neurons are more responsible for the output error that is caused by the defects, the gates are activated. On the contrary, if the other neurons are thought to be little related to the output error, the gates are deactivated during the retraining. By doing so, we can reduce the number of memristors that are to be programmed during the retraining. Obviously, if the number of memristors retrained is reduced, the time and power for programming memristors can also be decreased. The equation calculating each synaptic weight can be expressed with Equation (1).

(1) $$w_{\mathrm{ji},\mathrm{new}}=w_{\mathrm{ji}}-w_{j,\mathrm{DG}}\cdot \alpha \cdot \langle \frac{\partial E}{\partial w_{\mathrm{ji}}}\rangle$$

Here, w_ji_ means the present synaptic weight from neuron i to neuron j. w_ji,new_ is the synaptic weight to be updated. w_j,DG_ is the weight of neuron’s gate j. If the gate is activated, w_j,DG_ is 1. For the deactivation, w_j,DG_ is 0. In the gradient descent equation, E is the output error and α is the learning rate. < · > means the average value over a batch. To decide which gates are activated and deactivated, we have to calculate the backward error, δ_j_ of neuron j, for the memristor crossbar with real defects. The equation of δ_j_ is expressed with Equation (2) [22].

(2) $$\delta_{j}=\varphi_{j}^{\prime }\left({\mathrm{net}}_{j}\right)\underset{i\in P_{j}}{\sum }\delta_{i}\cdot w_{\mathrm{ij}}$$

Here, φ(·) is the activation function and net_j_ is the summation of input values for the neuron j in the crossbar. P_j_ is the set of neurons posterior to the neuron j. Once we calculate the backward error of neuron j, we can decide whether we activate or deactivate the gate of neuron j, with Equation (3).

(3) $$w_{j,\mathrm{DG}}=\{\begin{array}{c} 1\;\mathrm{if}\;\left|\delta_{j}\right|>\phi_{\mathrm{th}\_\mathrm{train}}\\ \;0\;\mathrm{otherwise}\; \end{array}$$

w_j,DG_ means the binary weight of the gate of neuron j and φ_th_train_ is the threshold for deciding the activation or deactivation of the gate. If the backward error for the neuron j is larger than the threshold, we think that the neuron j is more responsible for the output error and it should be reprogrammed in the retraining step. If the backward error, δ_j_ is smaller than the threshold, then we think that the neuron j is partially responsible for the output error.

### 2.3. The Partial-Gated Memristor Crossbar

**Figure 3:**
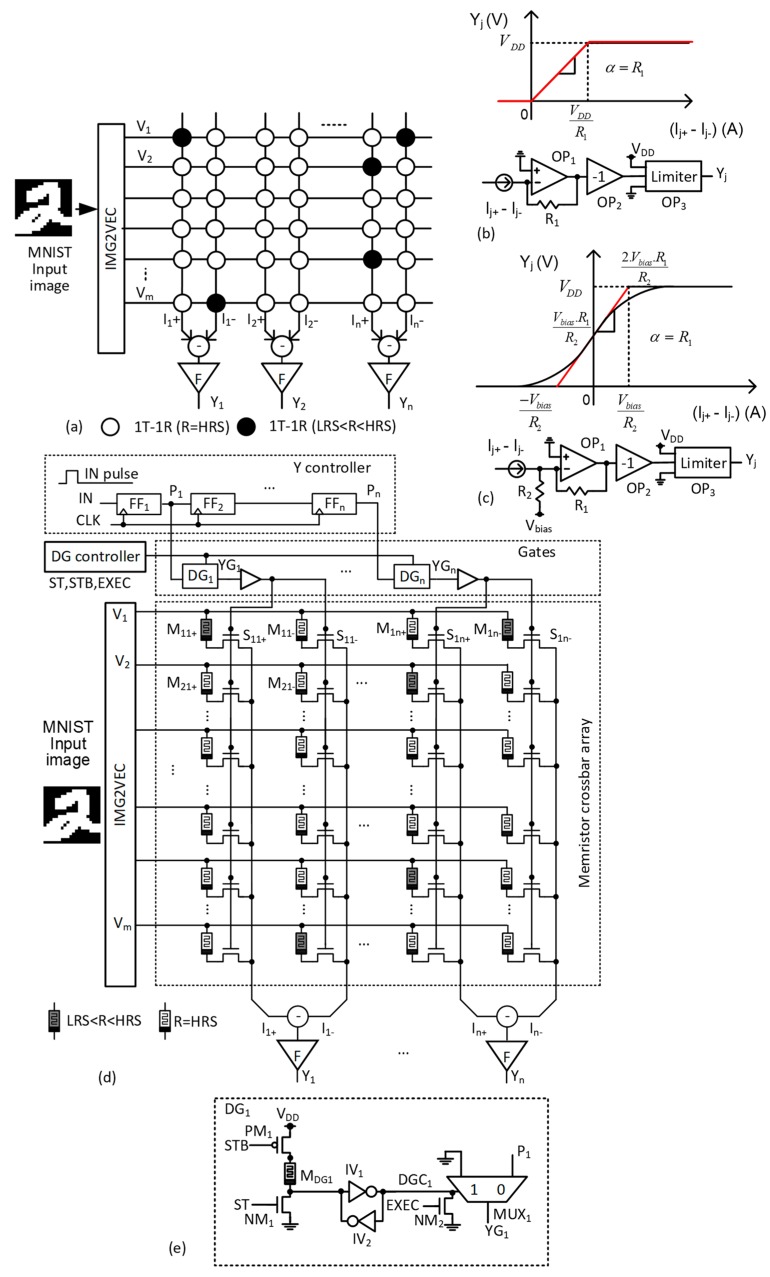
(**a**) The previous 1T-1R memristor crossbar without the partial gating, (**b**) the ReLU activation function circuit and its transfer curve [24], (**c**) the Sigmoid activation function circuit and its transfer curve [24], and (**d**) the proposed memristor crossbar circuit with the partial gating (**e**) the partial gating circuit for controlling the selectors.

Figure 3a shows the conventional memristor crossbar without the gates [23]. Here, V_0_, V_1_, etc. are the input voltages to the rows. If we assume the input vectors of 28 × 28, then the number of rows is as many as 784, as shown in Figure 3a. I_1+_ and I_1−_ are the positive current and negative current for the first column, respectively. $I_{+}-I_{-}$ is delivered to the following neuron with the activation functions, such as ReLU, Sigmoid, etc. Here, F means a neuron with the activation function. Y_0_, Y_1_, etc. represent the outputs of neurons. Each cell in the crossbar is assumed to have 1T and 1R. The memristor controlled by the transistor can be programmed by an intermediate value between HRS and LRS. Here, we can consider three cases of weights, which are positive, negative, and zero, respectively. For zero weight, both the (+)-column and (−)-column memristors should be programmed as HRS. By doing so, $I_{+}-I_{-}$ can be calculated with $I_{+}-I_{-}=(g_{\mathrm{HRS}}-g_{\mathrm{HRS}})\times V_{1}\approx 0$. g_HRS_ means HRS’s conductance. For the positive weight, the memristor of I+ should be between HRS and LRS. The memristor of I- should be HRS. If so, $I_{+}-I_{-}=(g_{m}-g_{\mathrm{HRS}})\times V_{1}$ is positive. g_m_ means the memristor’s conductance between HRS and LRS. For the negative weight, the memristor of I+ should be HRS and $I_{+}-I_{-}=(g_{\mathrm{HRS}}-g_{m})\times V_{1}$. Here, g_m_ is larger than g_HRS_.

Figure 3b,c show the activation function circuits and the transfer curves of ReLU and Sigmoid activation functions, respectively [24]. F means the activation function circuit. The ReLU circuit is used for the hidden neurons and the Sigmoid for the output neurons. In the ReLU circuit, OP_1,_ converts the input current of I_j+_ - I_j−_ to the voltage of −(I_j+_ − I_j−_) × R_1_. OP_2,_ is a simple inverting buffer, where –(I_j+_ − I_j−_) × R_1_ is inverted to (I_j+_ − I_j−_) × R_1_. OP_3_ acts as a limiter to keep the voltage within V_DD_ and ground potential (GND). If the output Y_j_ voltage is higher than V_DD_ or is lower than GND, the output voltage is limited by V_DD_ or GND, respectively. For the Sigmoid circuit, R_2_ and V_bias_ are used to realize the Sigmoid’s transfer curve by shifting the ReLU’s transfer curve by −V_bias_/R_2_. In Figure 3c, the black line represents the mathematical Sigmoid function and the red line indicates the transfer curve that is implemented by the Sigmoid circuit.

Figure 3d illustrates a partial-gated 1T-1R memristor crossbar circuit that is proposed in this paper. As described in Figure 3a, to receive 784 inputs from test vectors, the number of rows should be 784, which are represented by V_1_, V_2_ signal, etc. DG_1_ and DG_n_ represent the gates for controlling the 1st and nth columns, respectively. S_11+_ and S_11−_ are the selectors for M_11+_ and M_11−_ memristors, respectively. Here, the (+) and (−) symbols mean the positive and negative columns in the double-column architecture, respectively, which are used to calculate the positive and negative weights in the neural networks. The column current, I_1+_ is the positive summation of the weighted input vectors for the 1st column. Similarly, the column current, I_1−_ is the negative summation. I_n+_ and I_n−_ are the positive and negative summations of the nth column, respectively. The column currents are delivered to the activation function circuits of F, as shown in Figure 3d. For controlling the gates, the Y controller generates P pulses, such as P_1_, P_2_, etc. P_1_ and P_n_ are the pulses that are delivered to the 1st and nth columns in the crossbar in Figure 3d. The controller has the shift register chain, where the one-shot pulse is shifted by one column for one CLK cycle. When a column is partially responsible for the error, the gate for this column is deactivated during the retraining time. By doing so, we can keep this column from being programmed during the retraining.

Figure 3e shows the schematic of the partial gating circuit. ST and STB are delivered to the gates at the start-up of the retraining time. When ST and STB are high and low, respectively, NM_1_ and PM_1_ are turned on. If the memristor M_DG1_ has LRS, then DGC_1_ becomes ‘0’. In this case, the multiplexer delivers a signal P_1_ to the selectors. If the memristor M_DG1_ has HRS, DGC_1_ becomes ‘1’. The multiplexer delivers GND to turn off the selectors connected to DG_1_. By doing so, we can control the selector during the retraining time according to the programed M_DG_ value. On the contrary, during the execution time, all of the gates should be turned on. To do so, we added NM_2_ controlled by EXEC signal to Figure 3e. When the signal, EXEC becomes ‘1’ during the execution time, NM_2_ is on and the multiplexer delivers P_1_ to the selectors, regardless of the programmed M_DG1._

**Figure 4:**
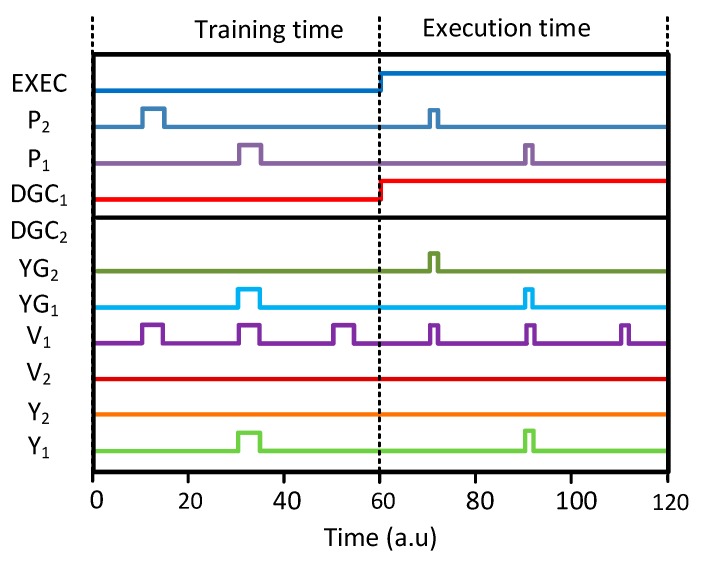
The simulated waveforms of the memristor crossbar with the parting gating.

The simulated waveforms illustrated Figure 4 demonstrate the operation of the memristor crossbar with the gates shown in Figure 3d,e. Here, the circuit simulation was performed using CADENCE SPECTRE and SAMSUNG 0.13-µm circuit-simulation parameters [25]. The Verilog-A model from the measured memristors was used in the circuit simulation. The Verilog-A model that was used here is explained in detail in the previous publication [11]. The operation of the partial-gated memristor crossbar is composed of the retraining time and execution time, as shown in Figure 4. During the retraining time, DGC_1_ and DGC_2_ are decided by M_DG1_ and M_DG2_, respectively. Here, we assume that M_DG1_ and M_DG2_ are LRS and HRS, respectively. If M_DG1_ are M_DG2_ are LRS and HRS, respectively, then DGC_1_ and DGC_2_ become low and high, respectively, during the retraining time. If so, MUX_2_’s output is fixed by GND and only MUX_1_ can deliver P_2_ to the selectors, as shown in Figure 4. In the execution time, EXEC signal can make both DGC_1_ and DGC_2_ low. Therefore, MUX_1_ and MUX_2_ deliver P_1_ and P_2_ to YG_1_ and YG_2_, respectively. As described in the previous section, the gates are only activated or deactivated during the retraining time to save the programming time and power. During the execution time, all of the gates are activated to perform the recognition. As no memristor is programmed during the execution time, we do not need to save the programming time and power in the crossbar.

## 3. Results

**Figure 5:**
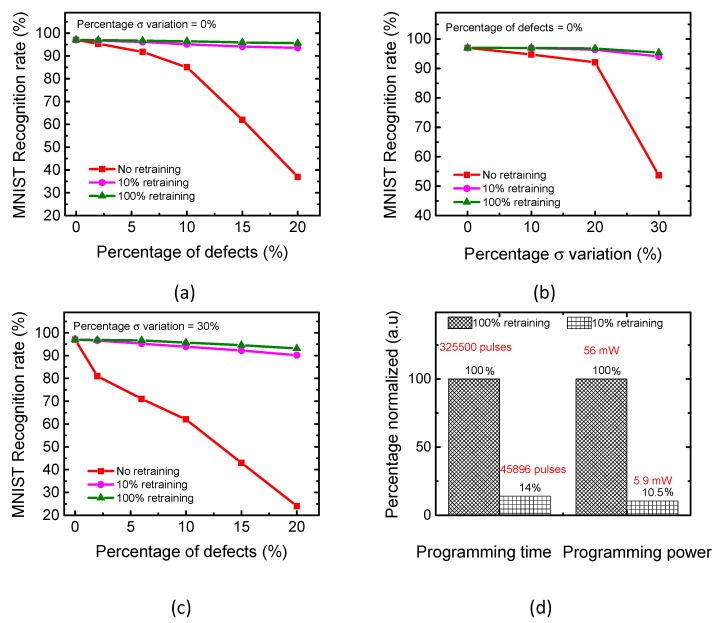
(**a**) The simulated MNIST recognition rates of the 0%, 10%, and 100% retraining with varying the percentage of stuck defects from 0% to 20% (**b**) the simulated MNIST recognition rates of the 0%, 10%, and 100% retraining with varying the percentage σ variation in memristor’s conductance (**c**) the simulated MNIST recognition rates considering both the stuck defects and percentage σ variation in weights (**d**) the programming time and power of the 10% and 100% retraining. In the programming time, we compared the total number of programming pulses between the 10% and 100% retraining.

For calculating the recognition rate, we tested the Modified subset of National Institute of Standards and Technology (MNIST) vectors [26,27] for the proposed partial-gated memristor crossbar. Here, MNIST stands for Mixed National Institute of Standards and Technology. The neural network that was tested in this paper has 100 hidden neurons and 10 output neurons. The 10 output neurons can distinguish 10 digits from 0 to 9. The number of input neurons is 784, because each MNIST vector is 28 × 28. Each MNIST vector is a gray image of 256 levels with 28 × 28 pixels. In the simulation, we programmed each memristor with 64 levels. In most of the experimental measurements, six-bit resolution with 64 levels can be thought to be the highest resolution of memristor’s programming [28]. As mentioned earlier, the memristors can have stuck-at-faults, such as stuck-at-on and stuck-at-off. Additionally, the variation-related defects can be observed in memristors. In Figure 5a, we varied the percentage of memristor defects in the crossbar from 0% to 20%. The 20% defects in the crossbar can be thought to be enough, because the previous publication experimentally reported that 11% of the 6912 memristors fabricated were the stuck-at-fault defects [29].

In Figure 5a, the rectangle symbols represent the recognition rate for no retraining. The circle and triangle symbols represent 10% and 100% retraining, respectively. The gap between the 10% and 100% retraining in Figure 5a is as small as 2.07%, for the percentage of defects = 20%. Here, the 10% retraining means that only the 10% of memristors in the crossbar are programmed during the retraining. The 100% retraining means that the entire crossbar is retrained. 

Figure 5b shows the recognition rate with varying the percentage σ variation in memristor’s conductance. In the simulation, it is assumed all of the synaptic weights in the crossbar are susceptible to the percentage σ variation that is changed from 0% to 30%. The previous publication reported that the standard deviation in memristor’s conductance was measured to be less than ~6µS for the target conductance ~100 µS [28]. This experiment clearly indicated that the real value of the percentage σ variation was observed at around ~6%, which could be thought to be much smaller than the percentage σ variation 30% in Figure 5b [28]. In Figure 5c, we varied the percentage of stuck defects from 0% to 20%, for the percentage σ variation as high as 30%. We can see that the 10% retraining can recognize the MNIST digits as well as the 100% retraining in Figure 5c.

Figure 5d compares the programming time between 10% and 100% retraining of the memristor-based network. For calculating the programming time, we used the transient behavior of memristor’s conductance dynamically changed according to the number of programming pulses, which was reported in the previous publication [14]. Here, the programming time can be calculated with the ‘m’ times the programming pulse width, as mentioned in Figure 1c. The number of pulses ‘m’ for programming the memristor’s conductance until the target weight is obtained from the relationship of memristor’s conductance and the number of pulses [14]. The Verilog-A model can model this relationship and it is used in the simulation in this paper. When comparing the 10% and 100% retraining, we can know that the programming time is proportional to the percentage of cells that are needed to be programmed in the memristor crossbar during the retraining. The 10% retraining means that only 10% of memristors are programmed during the retraining time, whereas all of the cells should be programmed in the 100% retraining. Thus, the 10% retraining of the crossbar can reduce the programming time significantly compared with the 100% retraining. For programming memristors, the ISPP scheme is used like Flash memories, as shown in Figure 1c, where the verifying pulse follows the programming pulse. Here, the programming and verifying pulse widths are 1 mS and 0.1 mS, respectively, in the program-verify scheme. From the simulation, the programming time for the 10% retraining is estimated to be shorter by 86% than the 100% retraining, as shown in Figure 5d, where the number of programming pulses is compared between the 10% and 100% retraining.

The programming power is also compared between the 10% and 100% retraining in Figure 5d. As expected, 10% retraining can reduce the power consumption by as much as 89.5% than the 100% retraining. The power overhead due to the partial gating circuit in Figure 3e is estimated to be very small, when compared to the power consumption of the memristor crossbar. Table 1 compares the power consumption of the 100% and 10% retraining for different LRS and HRS values. In addition, the power consumption of the memristor array is compared to the overhead power due to the partial gating circuit in Figure 3e. From this table, we can know the overhead power that is due to the gating circuit is negligible, even for the very high memristance with LRS = 1 MΩ and HRS = 100 MΩ, as compared to the crossbar’s power consumption. The overhead power in Table 1 includes the power consumption of the pulse generator and the partial gating circuit in Figure 3d,e. However, the power consumption of the gate controller for generating the control signals, such as ST, STB, EXEC, etc. is not included in Table 1. In Figure 3d, the gate controller can be shared among all of the crossbars in the entire chip. Thus, the overhead power due the gate controller can be neglected in this power calculation, as compared to the crossbar’s power consumption in Table 1. 

**Figure 6:**
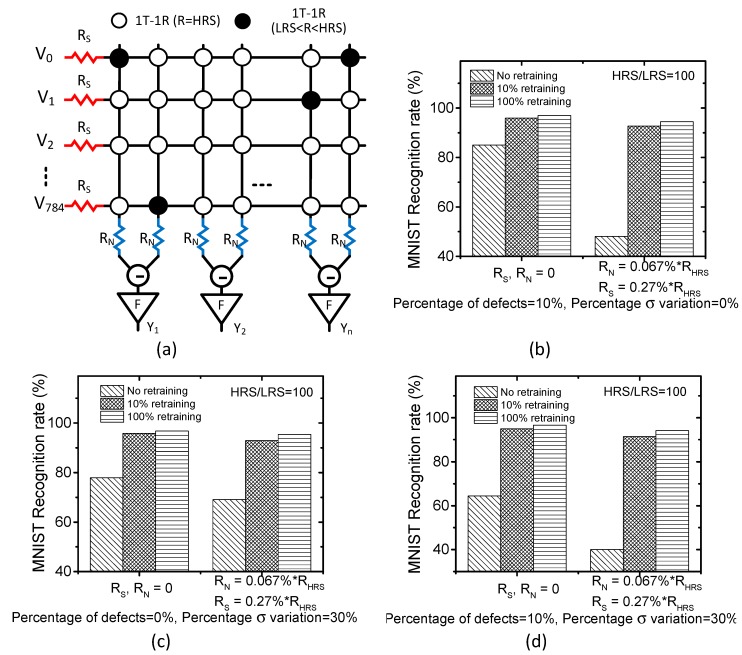
(**a**) The schematic of memristor crossbar with non-ideal source and neuron resistance. Here R_S_ and R_N_ represent source resistance and neuron resistance, respectively. (**b**) The MNIST recognition rates of the ideal (R_N_ = R_S_ = 0) and non-ideal (R_N_ = 0.067% of R_HRS_, R_S_ = 0.27% of R_HRS_) crossbars for the percentage of stuck defects = 10% and the percentage σ variation in weights = 0% (**c**) the MNIST recognition rates of the ideal (R_N_ = R_S_ = 0) and non-ideal (R_N_ = 0.067% of R_HRS_, R_S_ = 0.27% of R_HRS_) crossbars for the percentage of stuck defects = 0% and the percentage σ variation in weights = 30% (**d**) the MNIST recognition rates of the ideal (R_N_ = R_S_ = 0) and non-ideal (R_N_ = 0.067% of R_HRS_, R_S_ = 0.27% of R_HRS_) crossbars considering both the percentage of stuck defects = 10% and the percentage σ variation in weights = 30%.

In the real memristor crossbar, we need to consider the non-ideal effects, such as source resistance, neuron resistance, etc. [30]. Figure 6a shows the schematic of memristor crossbar that includes the non-ideal source and neuron resistance. Here, R_S_ and R_N_ represent source resistance and neuron resistance, respectively [30]. Figure 6b compares the recognition rates between the ideal and non-ideal crossbars for the 0%, 10%, and 100% retraining. The comparison is performed with the percentage of stuck defects = 10% and the percentage σ variation in weights = 0%. In Figure 6b, the ideal crossbar has R_N_ = R_S_ = 0. In the non-ideal crossbar, R_N_ and R_S_ are assumed to be 0.27% of R_HRS_ and 0.067% of R_HRS_, respectively [30]. R_HRS_ means the resistance value of High Resistance State. These R_S_ and R_N_, which are 0.27% of R_HRS_ and 0.067% of R_HRS_, respectively, are the worst-case values of the source and neuron resistance observed from the real crossbars fabricated [30]. From Figure 6b, we can see the partial learning can also be applied to the non-ideal crossbar as well as the ideal crossbar. Figure 6c compares the ideal and non-ideal crossbars when the percentage of stuck defects = 0% and the percentage σ variation in weights = 30%. Figure 6d shows the recognition rates when the percentage of stuck defects = 10% and the percentage σ variation in weights = 30%.

**Figure 7:**
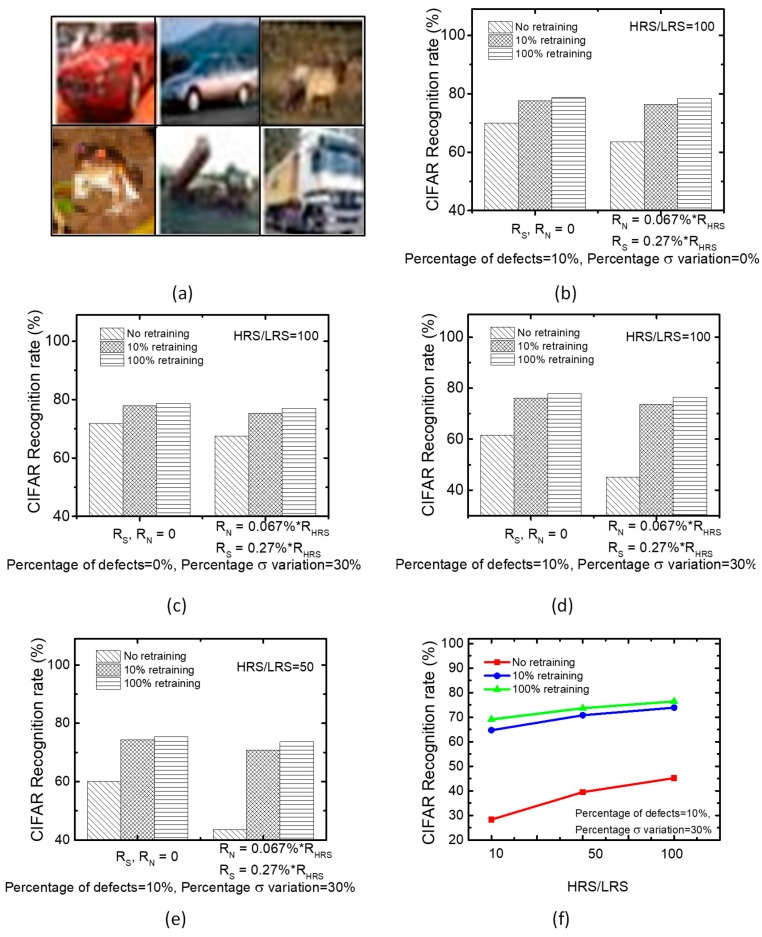
(**a**) The CIFAR-10 image samples (**b**) The CIFAR-10 recognition rates of the ideal (R_N_ = R_S_ = 0) and non-ideal (R_N_ = 0.067% of R_HRS_, R_S_ = 0.27% of R_HRS_) crossbars for the percentage of stuck defects = 10% and the percentage σ variation in weights = 0%.Here, HRS/LRS = 100. (**c**) The CIFAR-10 recognition rates of the ideal (R_N_ = R_S_ = 0) and non-ideal (R_N_ = 0.067% of R_HRS_, R_S_ = 0.27% of R_HRS_) crossbars for the percentage of stuck defects = 0% and the percentage σ variation in weights = 30%. Here, HRS/LRS = 100. (**d**) The CIFAR-10 recognition rates of the ideal (R_N_ = R_S_ = 0) and non-ideal (R_N_ = 0.067% of R_HRS_, R_S_ = 0.27% of R_HRS_) crossbars when considering both the percentage of stuck defects = 10% and the percentage σ variation in weights = 30%. Here, HRS/LRS = 100. (**e**) The CIFAR-10 recognition rates of the ideal (R_N_ = R_S_ = 0) and non-ideal (R_N_ = 0.067% of R_HRS_, R_S_ = 0.27% of R_HRS_) crossbars considering the percentage of stuck defects = 10% and the percentage σ variation in weights = 0%. Here, HRS/LRS = 50. (**f**) The CIFAR-10 recognition rate with varying HRS/LRS ratio from 10 to 100.

The proposed training can also be applied to CIFAR-10 dataset with various color images [31]. The simple MLP-based networks are not suitable for solving the recognition task of CIFAR-10, which is shown in Figure 7a. Therefore, we employed the LeNet CNN architecture instead of using the simple MLP architecture for testing CIFAR-10. First, the convolution layers on the CNN are utilized to learn the key features of CIFAR-10 dataset. Subsequently, the extracted features are delivered to the following MLP network in order to classify the items of CIFAR-10. By applying the same conditions as Figure 6, we evaluated the real crossbar’s recognition performance with the non-ideal parameters. The loss of the recognition rate in the non-ideal crossbar is compared between the 10% and 100% retraining, in this paper. The loss is observed to be very small for Figure 7b–d, respectively. Here, we also assumed R_S_ = 0.27% of R_HRS_ and R_N_ = 0.067% of R_HRS_, respectively, for considering the non-ideal parameters of the real crossbar [30]. Here, the HRS/LRS ratio is fixed by 100 for Figure 7b–d. In addition, we tested the CIFAR-10 recognition rate for HRS/LRS ratio = 50 in Figure 7e. The result with HRS/LRS = 50 is very similar with the simulation with HRS/LRS = 100. Moreover, we tested the recognition rate for HRS/LRS ratio = 10, as in Figure 7f. 

When considering the non-ideal R_S_ and R_N_, the recognition rate of the 10% retraining is lowered by 2.9% than the 100% retraining, for a worst-case condition of HRS/LRS = 50, the percentage of stuck defects = 10% and the percentage σ variation in weights = 30%, as shown in Figure 7e. In terms of the accuracy-sensitive scenario, a rate-loss as large as 2.9% can be considered to be significant. However, some neural network’s applications such as Quantization Neural Network (QNN) may focus on the simple and fast computation more than the network’s recognition performance [32,33,34,35]. Usually, for the edge-computing applications, the power and time should be seriously considered. In this regard, the proposed partial gating scheme that can significantly save the training time and power may be useful in the neural network applications of edge computing, where the fast training with high energy-efficiency is very seriously considered.

## 4. Discussion

The first thing to discuss here is that the rate-loss as large as 2.9% in Figure 7e can be better improved by the technology-aware training scheme, where the non-ideal R_S_ and R_N_ values, the percentage of stuck defects, the percentage σ variation in weights, etc. are considered to be technology parameters that can be managed to achieve the target accuracy [30]. The technology-aware training method can be used in the partial gating scheme to improve the rate-loss better, in order to reach the target accuracy of the neural networks. For example, if we can reduce the percentage of stuck defects from 10% to 5%, the rate-loss can be lowered from 2.9% to 1.5%. 

**Figure 8:**
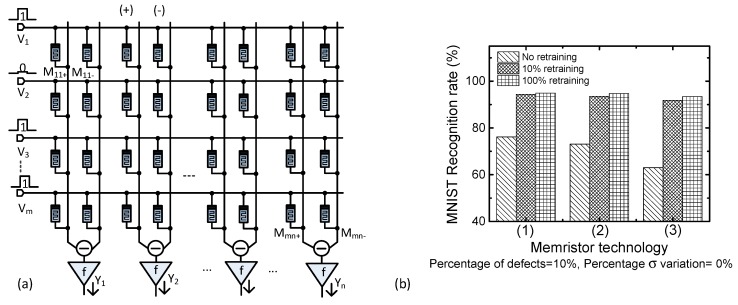
(**a**) Memristor Binarized NN with double-column crossbar, where memristors should be HRS and LRS not allowing intermediate values between HRS and LRS (**b**) the MNIST recognition rates of no-retraining, 100% retraining, and 10% retraining for different memristor technologies, refered in [1,11,12]. The technology (1) means the memristor measured in Figure 1b [11]. The technology (2) is from the previous publication [12]. The technology (3) is from the fabricated memristors [1].

One more thing to note here is that the rate loss due to the partial gating scheme can be compared to the state-of-the-art CMOS-implemented QNN, which was developed to replace the high-precision computation with the low-precision one [32]. Using the low-precision multiplication can result in 4~6X speed-up, in spite of roughly ~1–3% loss of recognition performance [32,33,34]. This ~1–3% loss is comparable to the rate loss that is due to the partial gating scheme, as shown in Figure 7 and Figure 8. Additionally, 4~6X speed-up of the CMOS-implemented QNN can be compared with ~7X faster training time of the partial gating scheme with the 10% retraining [33]. The energy efficiency of the CMOS implemented QNN is ~3.1X better the high-precision network [35]. Thus, comparing the partial gating scheme with the state-of-the-art CMOS-implemented QNN indicates that the proposed scheme can be very useful for energy-efficient and fast training for the edge-computing neural networks, like QNN.

For the comprehensive comparison of the loss of recognition rate, training time, and training power, the partial gating scheme is compared with the other memristor-crossbar-based neural networks and CMOS-based QNN, as in Table 2. The four memristor-crossbar-based schemes and one CMOS-based QNN in Table 2 can be thought to be suitable to the neural networks for edge-computing applications. The compared schemes are as follows. (1) The memristor crossbar that was implemented from the six-bit Neural Network (NN) with 100 retraining of the entire network. (2) The memristor crossbar implemented from the network’s pruning algorithm [18,19]. (3) The memristor crossbar implemented from QNN algorithm [32,33,34] (Memristor Binarized NN [24]). (4) The proposed partial gating scheme. (5) The CMOS-based QNN [35].

Actually, the network pruning and QNN were developed to compress the network size and to avoid the complicated multiplication, respectively, not to reduce the training time and power [18,19,32,33,35]. However, if we implement the network pruning with the memristor crossbar, then we can save the training time and power of the memristor crossbar, like the partial gating scheme. For the network pruning, we reduced the network size by 90% by eliminating the insignificant neurons. Thus, only the remaining 10% neurons are retrained after the network pruning. It means that the training time for the 90%-pruned network can be ~8X faster than the 0%-pruned network. In Table 2, we assumed the same defect map with the same percentage of defects for comparing the four memristor-crossbar-based schemes of the columns (1)–(4), respectively. Additionally, we assumed the non-ideal parasitic resistance values R_S_ = 0.27%-R_HRS_ and R_N_ = 0.067%-R_HRS_ that are the same values with Figure 6 and Figure 7. For simulating the memristor-crossbar-implemented QNN, we used the binary synaptic weights of memristor crossbar [24]. By doing so, we could decrease the rate-loss as low as ~4% in the memristor-crossbar-implemented QNN. However, it should be noted that the memristor QNN could not save the training time, unlike the partial gating scheme. This is because the entire memristor crossbar should be trained in the memristor-crossbar-implemented QNN, such as Memristor Binarized NN [24]. The advantage of memristor QNN is that programming memristors with HRS and LRS can be more reliable than the programming analog or multi-valued memristors [24].

From Table 2, in terms of the training time, training power, and loss of recognition rate, the proposed partial gating scheme (4) shows the best performance among all of the schemes. The loss of recognition rate for the partial gating scheme is as low as ~2% and the training time can be ~7X faster than the memristor crossbar that was implemented from six-bit NN with 100% retraining. The training power of the proposed partial gating scheme (4) is ~7X smaller than the 100% retraining scheme (1). This is because only the parts of memristors need to be programmed in the partial gating scheme, as already explained. 

The training power of the memristor crossbar of QNN (3) is ~2.1X larger than the six-bit NN crossbar (1) in Table 2, though both the QNN and 6-bit NN crossbars have the same training time. Actually, the training power depends on the number of LRS cells that cause a larger amount of programming current than HRS cells. The QNN crossbar (3) has more LRS cells than the six-bit NN crossbar (1), resulting in the larger training power of the QNN crossbar (3) than (1), as shown in Table 2. The training power of the pruned crossbar (2) is almost the same as the proposed partial-gated crossbar (4).

One more thing to note here is that the proposed partial gating scheme can be applied to the memristor-crossbar-based Binarized NN [24]. Figure 8a shows a memristor binarized NN with the binary synaptic weights of +1, 0, and −1 [24]. Here V_1_, V_2_, etc. represent the input voltages entering the crossbar. V_1_, V_2_, etc. are connected with the output neurons of Y_1_, Y_2_, etc. through the memristor connections of M_11+_, M_11__−_, etc. In Figure 8a, M_11_+ and M_11_- represent the positive and negative synaptic connections, for the (+) and (−) columns, respectively. They should be HRS and LRS not allowing any intermediate state between HRS and LRS in Figure 8a. Here, the (+) and (−) columns are used to consider the positive and negative synaptic weights, respectively. The symbols that are denoted as f mean the activation function circuits, where the column current calculated from the positive and negative columns is converted to a voltage according to the activation function. One thing to note in Figure 8a is that no transistor is used as a selector in the binary memristor crossbar. The previous publication experimentally showed that the binary memristor crossbar could be built without transistor as a selector [36]. In the binary crossbar, HRS and LRS are programmed while using the V_DD_/3 method to minimize the write disturbance problem [36].

Figure 8b compares the MNIST recognition rates of no-retraining, 100% retraining, and 10% retraining for different memristor technologies. Here, the percentage of memristor defects is assumed to be 10%. In Figure 8b, the technology (1) is for the memristor technology in Figure 1b. The technology (2) is from the previous publication [12]. The technology (3) is from the publication [1]. Figure 8b shows that the 10% retraining of the memristor crossbar can recover the recognition rate loss due to the memristor defects as well as the 100% retraining. This result strongly indicates that the partial gating scheme can be used in not only the multi-valued, but also in the memristor binarized NNs, for various fabrication technologies of real memristors.

In Figure 8b, we only trained the parts of binary memristors chosen, according to the error calculation in Equations (2) and (3), instead of training the entire crossbar. By doing so, we could reduce the total number of programming pulses for significantly training the binary memristor crossbar. When considering the programming speed, we may think the binary memristor can be programmed very fast by a single or double pulses without using the program-verify scheme, such as ISPP, unlike the multi-valued memristor [13,37]. However, if we do not use the program-verify scheme for training the binary memristor, the programming precision becomes much worse [14]. The number of programming pulses in the program-verify scheme severely affects the programmed HRS and LRS statistical distributions of the binary memristor crossbar, as experimented in the previous publication [14]. If we want to program the binary memristor with a fine precision, the number of programming pulses should be, for example, as many as 30 [14]. By doing so, we can control the statistical distributions of LRS and HRS as narrow as the percentage σ variation = 5% [14]. If we program the binary memristor with a coarse precision, the number of programming pulses can just as small as 3 [14]. In this coarse programming, the statistical distributions of LRS and HRS were measured as wide as the percentage σ variation = 30% [14]. This wide σ of the coarse programming of HRS and LRS can degrade the MNIST recognition rate very much, as low as 57% [14]. Thus, it should be noted here that even the binary memristor crossbar needs the fine programming scheme, where the large number of programming pulses should be used in the fine program-verify method. The proposed partial gating scheme can reduce the total number of programming pulses for the binary memristor crossbar, as it does for the multi-valued memristor crossbar. Thus, the reduced number of programming pulses can result in fast and energy-efficient training, not only for the multi-valued, but also for the binary memristor crossbar.

One concern is that the recognition rate of binary-memristor-based networks would be worse than the multi-valued ones. Table 2 clearly indicates this degradation, for the memristor binarized NN that used the synaptic weights of only LRS and HRS. Recently, the multi-valued memristor crossbars have been observed to be capable of having more than six-bit resolution (= 64 levels) [28,37,38]. When comparing the commercial neural network chips with high precision, the six-bit precision still seems lower [39]. However, the multi-valued crossbars have been tested to show very good performance in MNIST recognition, DCT image processing, etc., in spite of six-bit [28,38,40]. Thus, we can think that the binary memristor crossbars can be useful in some applications that demand low performance. On the contrary, we should use the multi-valued memristor crossbars, such as six-bit, in spite of high variability and low error-resilience, as mentioned in the previous publications, for high performance [28,37,38]. To mitigate the demerits of multi-valued computation, some algorithmic techniques, such as Error Correction Coding (ECC) can be considered in memristor-crossbar-based networks. Actually, ECC has been common in modern state-of-the-art memory technologies, such as DRAM, FLASH, etc., regardless of the binary and multi-valued circuits, for many years [41].

One more concern in the memristor crossbar’s training is the scalability issue for realizing a large-size memristor array. In terms of the scalability, the partial gating scheme can be very good, because the proposed scheme is based on 1T-1R crossbar architecture, where only one column is activated at one moment and the rest of columns are turned off. This column-by-column activation in the crossbar can significantly reduce an amount of sneak leakage, resulting in eliminating the disturbance problem that is caused from the neighbor’s sneak leakage current. Moreover, the programming time and power for training the crossbar can be significantly reduced in the partial gating scheme, as compared to the 100% retraining scheme. The time and power reduction in the crossbar’s training will be very useful in the edge-computing applications of nanoscale memristor-based neural networks [14]. However, the stuck-at-fault and variation defects still threaten the scalability of memristor crossbars, hindering the memristor crossbar from being fabricated in a large array, and limiting the memristor array size less than ~10K cells [28].

## 5. Conclusions

Real memristor crossbars can have defects, which should be considered in training the memristor-based neural networks. Training the memristor crossbar refers to programming memristors according to the calculated synaptic weight using the back-propagation algorithm. Unfortunately, programming the crossbar takes a very long time and consumes a large amount of power, because of the incremental behavior of memristor’s program-verify scheme for the fine-tuning of memristor’s conductance to achieve a high recognition rate. To reduce the programming time and power in the crossbar’s training, the partial gating scheme was proposed in this paper, where the neurons were divided into two groups, according to the neurons responsible or not for the output error. Training only parts of neurons that are responsible to the output error can significantly reduce the programming time and power of memristors, as compared to training the entire crossbar.

CADENCE circuit simulation with the Verilog-A model of the memristors obtained from the previous publications verified the proposed scheme. The recognition rate was simulated with MNIST and CIFAR-10 datasets for the ideal and non-ideal crossbars with non-ideal parameters, such as R_S_, R_N_, etc. When comparing the 100% and 10% retraining, the loss of recognition rate was observed only as small as 2.5% and 2.9%, for MNIST and CIFAR-10 datasets, respectively. However, the programming time and power of the 10% retraining could be reduced by as much as 86% and 89.5%, respectively, when compared to the 100% retraining.

## Figures and Tables

**Table 1:** Comparison of power consumption of 10% and 100% retraining for different LRS and HRS. In addition, the overhead power due to the partial gating circuit in Figure 3e is compared to the power consumption of the memristor array with selectors. The comparison in Table 1 indicates the overhead power is negligibly small than the array power consumption.

HRS/LRS ratio = 100	The power consumptions of the memristor array and selector for 100% retraining	The power consumptions of the memristor array and selector for 10% retraining	Overhead power due to the power gating circuit for 10% retraining
LRS = 10 kΩ HRS = 1 MΩ	56,000 µW	5910 µW	1.6 µW
LRS = 100 kΩ HRS = 10 MΩ	7140 µW	670 µW	1.1 µW
LRS = 1 MΩ HRS = 100 MΩ	645 µW	69 µW	0.86 µW

**Table 2:** Comparison of the loss of MNIST recognition rate, training time, and training power, among four memristor-crossbar-based schemes and one CMOS-based Quantization Neural Network (QNN). The five schemes are as follows. (1) The memristor crossbar implemented from the 6-bit NN with 100%-retraining. (2) The memristor crossbar implemented from the network’s pruning algorithm, refered in [18,19]. (3) The memristor crossbar implemented from QNN algorithm, refered in [32,33,34], (Memristor Binarized NN [24]). (4) The proposed partial gating scheme. (5) CMOS-based QNN, refered in [35]. In the crossbar simulation with MNIST dataset, the same defect map and the same percentage of defects = 10% are assumed. The non-ideal parasitic resistance values are R_S_ = 0.27%-R_HRS_ and R_N_ = 0.067%-R_HRS_ that are the same values with Figure 7 and Figure 8.

Scheme	(1)	(2)	(3)	(4)	(5)
Implementation	Memristor crossbar				CMOS
Rate-loss	~0%	~10%	~4%	~2%	~1-3% compared to the full-precision CMOS NN
Training time	~1X	~8X faster	~1X	~7X faster	~4-6X faster than the full-precision CMOS NN
Training power	~1X	~10X smaller	~2.1X larger	~10X smaller	~3.1X smaller than the full-precision CMOS NN
